# 759 Burn Care During the COVID-19 Pandemic: Assessing the Current Literature

**DOI:** 10.1093/jbcr/irac012.312

**Published:** 2022-03-23

**Authors:** Stephen P Tranchina, Justin Tran, Elias A Ghafoor, Lauren T Moffatt, Tina L Palmieri, Jeffrey W Shupp, Laura S Johnson, Kathleen S Skipton Romanowski

**Affiliations:** Firefighters’ Burn and Surgical Laboratory at MedStar Washington Hospital Center, Arlington, Virginia; University of California, Davis, Union City, California; Firefighters’ Burn and Surgical Laboratory at MedStar Washington Hospital Center, Centreville, Virginia; Burn Center at MedStar Washington Hospital Center, Washington DC, District of Columbia; UC Davis and Shriners Hospitals for Children Northern California, Sacramento, California; MedStar Washington Hospital Center, Washington DC, District of Columbia; MedStar Washington Hospital Center / Georgetown School of Medicine, Washington, District of Columbia; UC Davis, Sacramento, California

## Abstract

**Introduction:**

The emergence of SARS-CoV-2 and the subsequent COVID-19 pandemic has been a significant disruptor to traditional medical care. Burn patients are an interesting population in which to evaluate this disruption due to the complicated, multidisciplinary nature of injury management. Understanding the current landscape of burn care during the pandemic is a crucial first step in preparing for future pandemic impacts. The purpose of this study was to identify the current status of burn treatment during COVID by evaluating existing literature surrounding burns and COVID.

**Methods:**

A literature review of articles published between March 2020 and August 2021 was conducted to determine trends in studies evaluating burn patients and burn center operation during this time frame. All ABA abstracts published in 2020 containing the key words “burn,” “COVID,” and/or “coronavirus” were reviewed. Additionally, a Pubmed search was conducted using the same keywords. Each abstract and article was sorted into one of four themes: Census/Etiology, Burn patients with COVID, Safe Practices/Protocols, and Telemedicine.

**Results:**

A total of 23 ABA abstracts and 126 articles were collected in the initial search. 63 articles were ultimately excluded because they did not report on burn patients. By theme, the following trends were seen: ***1. Census/Etiology:*** Data on demographics of burn patients during this period was varied. Admissions for adult and/or pediatric burns increased for multiple burn centers, while others reported decreases. Consistently, the most common etiology of burn injury was scald, and an increased proportion of injuries were found to occur at home. Changes in the rates of first, second, and third-degree burns were also observed. ***2. Burn patients with COVID***: Overall, numerous reports indicated decreases in patient length of stay. However, several groups found no differences in length of stay, surgery rate, and length of follow-up. ***3. Safe Practices*:** A recurring trend was observed of numerous burn centers having to implement increased safety protocols due to COVID-19. Select burn centers updated prevention guidelines for burn surgeons and patient care. ***4. Telemedicine: ***The implementation of telemedicine helped minimize risk and maximize resources, However, much remains to be standardized, including the quality of images used.

**Conclusions:**

This analysis of the current literature identified several overarching themes in the care of burn patients. Continued evaluation can identify innovations from the past year that should become best practices, as well as optimize preparation efforts for future disruptions in care.

